# Expressive language sampling and outcome measures for treatment trials in fragile X and down syndromes: composite scores and psychometric properties

**DOI:** 10.1038/s41598-023-36087-3

**Published:** 2023-06-07

**Authors:** Leonard Abbeduto, Laura del Hoyo Soriano, Elizabeth Berry-Kravis, Audra Sterling, Jamie O. Edgin, Nadia Abdelnur, Andrea Drayton, Anne Hoffmann, Debra Hamilton, Danielle J. Harvey, Angela John Thurman

**Affiliations:** 1 MIND Institute and Department of Psychiatry and Behavioral Sciences, University of California Davis Health, 2828 50Th St., Sacramento, CA 95817 USA; 2 Department of Neurology, Rush University Medical Center, Chicago, IL USA; 3 Waisman Center and Department of Communication Sciences and Disorders, University of Wisconsin-Madison, Madison, WI USA; 4 Department of Psychology, Sonoran UCEDD, UA Family and Community Medicine, University of Arizona, Phoenix, AZ USA; 5 Department of Communication Disorders and Sciences, Rush University, Chicago, IL USA; 6 Department of Pediatrics, Rush University, Chicago, IL USA; 7 Department of Human Genetics, Emory University School of Medicine, Atlanta, GA USA; 8 Department of Public Health Sciences, University of California, Davis, USA

**Keywords:** Psychology, Signs and symptoms

## Abstract

The lack of psychometrically sound outcome measures has been a barrier to evaluating the efficacy of treatments proposed for core symptoms of intellectual disability (ID). Research on Expressive Language Sampling (ELS) procedures suggest it is a promising approach to measuring treatment efficacy. ELS entails collecting samples of a participant’s talk in interactions with an examiner that are naturalistic but sufficiently structured to ensure consistency and limit examiner effects on the language produced. In this study, we extended previous research on ELS by analyzing an existing dataset to determine whether psychometrically adequate composite scores reflecting multiple dimensions of language can be derived from ELS procedures administered to 6- to 23-year-olds with fragile X syndrome (*n* = 80) or Down syndrome (*n* = 78). Data came from ELS conversation and narration procedures administered twice in a 4-week test–retest interval. We found that several composites emerged from variables indexing syntax, vocabulary, planning processes, speech articulation, and talkativeness, although there were some differences in the composites for the two syndromes. Evidence of strong test–retest reliability and construct validity of two of three composites were obtained for each syndrome. Situations in which the composite scores would be useful in evaluating treatment efficacy are outlined.

## Introduction

One in every 77 children in the U.S. has been diagnosed with an intellectual disability (ID)^1^. Advances in genetic science have led to the discovery of the etiologies of half of all cases of ID^2^. Research focused on uncovering commonalities and differences in the phenotypes of conditions resulting from disparate etiologies has led to etiology-specific behavioral and pharmacological treatments^3–7^. Although treatments have been tested for a range of ID conditions, pharmaceutical agents targeting symptoms of fragile X syndrome (FXS) and Down syndrome (DS) have been among the most frequently tested^8–10^. FXS is the leading inherited cause of ID, with a prevalence of 1 in 3600 males and 1 in 4000–6000 females^11–13^. DS is the leading genetic cause of ID, with a prevalence estimated at 1 in 691 live births^14^. In addition to ID, individuals with FXS or DS often demonstrate clinical features that overlap with other frequently occurring psychiatric and developmental conditions such as autism spectrum disorder (ASD), anxiety, and attention-deficit/hyperactivity disorder (ADHD)^15–18^. Moreover, virtually all individuals with FXS or DS display delays in language relative to chronological age expectations, and language lags cognitive development in many affected individuals.

Despite the growth of clinical trials for ID, studies have generally failed to demonstrate the efficacy of treatments targeting core symptoms. In the case of FXS, more than two dozen clinical trials testing a range of promising pharmaceutical therapeutics have largely failed to show efficacy^8^. Disappointing results from clinical trials for individuals with DS have also occurred^19,20^. The lack of adequate outcome measures to assess therapeutic efficacy in individuals with ID conditions is thought to be an important factor in the failure of these trials^4,21,22^. In particular, many of these trials have relied on parent report measures that produced large placebo effects or respondent bias^23^. Such effects and biases may obscure the benefits of the tested therapeutics^24,25^. Although norm-referenced standardized tests offer a more direct and objective assessment of change compared to informant-report measures, few such standardized tests have been validated as outcome measures for FXS, DS, or other conditions associated with ID^26^. In fact, the closely spaced, repeated administration of a measure required for most clinical trials is discouraged for most standardized tests because of concerns about practice effects and the short-term instability of scores^26^. Further, many individuals with ID often score at the floor of standardized tests, making it difficult to assess change from baseline^27,28^.

In response to the failure of so many clinical trials, there have been several recent programs of research designed to evaluate the psychometric adequacy of measures of various core symptoms of ID, with particularly promising measures emerging for several domains of cognition and behavior (e.g.,^29–31^). For example, impairments in expressive language are invariably associated with ID and adversely affect daily functioning^32,33^. Moreover, the expressive language skills of individuals with ID, including those with FXS or DS, are frequent intervention targets. There is, therefore, a pressing need for outcome measures in this domain^34^. Expressive Language Sampling (ELS) appears particularly promising in this regard^35^.

ELS entails collecting samples of the participant’s talk in naturalistic interactions with a partner^36^. In the version of the ELS procedures we developed^9,37^, the partner is an examiner. The format, content, and examiner’s behavior are scripted to ensure consistency across participants, measurement occasions, and examiners. The interactions, however, remain naturalistic; the examiner adapts (within strict constraints established by the scripts) to the interests and behavior of the participant. Audio-recordings of these samples are transcribed and analyzed using computer-based algorithms to derive outcome measures reflecting several dimensions of language ability, such as vocabulary and syntax. Finally, we developed procedures for collecting samples in two contexts—conversation and narration—with each context associated with different processing demands and social expectations.

ELS procedures have several potential advantages over current norm-referenced standardized tests of language. First, the interactions in which samples are collected in ELS are more similar to real-world, communicative contexts than is true for standardized tests, with the latter typically relying on visual or verbal prompts to elicit a target utterance rather than to communicate information to a partner^37^. As a result, ELS performance is more likely to be generalizable to activities that are functional and meaningful for the individual, which is a desirable property for an outcome measure from the perspective of regulatory bodies such as the U.S. Food and Drug Administration^36^. Second, the ELS contexts used in the present study can be used with children and adults as well as having low rates of noncompliance and limited floor effects for individuals producing at least some multiword utterances^9,37^. In contrast, many standardized tests are applicable only to a narrow age range or they use different types of items or testing formats for different age ranges. Third, numerous dependent measures, each reflective of a different dimension of language, can be computed from a single, brief expressive language sample^36,37^. The conversation and narration contexts of the present study require, on average, 10 and 20 min of participant time, respectively. In contrast, multiple standardized tests or a battery of subtests selected from multiple standardized tests would be needed to assess the same constructs, resulting in a much greater burden on the participant. Fourth, many standardized tests underestimate the language capabilities of children from ethnic and racial minorities relative to more naturalistic ELS procedures, which raises concerns about the sensitivity of the former in characterizing the true benefits of a treatment for minoritized participants^38^. Finally, intervention studies targeting children with various forms of ID have shown change in variables derived from ELS-type procedures despite a lack of change in their performance on various standardized tests^39–41^.

Although ELS procedures of various sorts have been used for decades in research and clinical practice, there has been little psychometric evaluation of the resulting measures for ID populations. Recently, however, Abbeduto et al.^37^ evaluated the psychometric properties of their manualized and highly scripted ELS conversation and narration procedures for 6- to 23-year-old verbal individuals with FXS and co-occurring ID. It was found that participants with FXS demonstrated noncompliance rates of less than 15% on both the conversation and the narration procedures. Five measures were derived for each sample, with the measures indexing maturity in talkativeness, unintelligibility of speech, fluency in planning, vocabulary, and syntax. Minimal practice effects and strong test–retest reliability were observed for the five measures across a 4-week interval. Furthermore, strong evidence of convergent construct validity was observed for the ELS measures of vocabulary, syntax, and unintelligibility. Additional evidence of construct validity for the vocabulary, syntax, and unintelligibility measures was obtained from a sample of 5- to 36-year-olds with FXS in the form of correlations with verbal and nonverbal IQ scores^35^. In addition, the ELS-derived measure of unintelligibility correlates with brain activity (reflected in EEG indices) during speaking^42^, providing further evidence of construct validity and suggesting that at least one ELS measure reflects impaired brain function. It has also been shown that several of the ELS measures correlate with the level of daily living skills, capacity for independent functioning, and self-determination for adolescents and young adults with FXS^33^. Finally, Thurman et al.^9^ extended the ELS measures to individuals with DS and with similarly compelling evidence of their psychometric adequacy as outcome measures.

We focused previous psychometric analyses of our ELS procedures on five variables conceptualized as representing separate dimensions of expressive language skill: speech articulation (proportion of unintelligible speech), vocabulary (number of different word roots), syntax (mean length of utterance in morphemes), planning (proportion of speech containing dysfluencies. i.e., repetitions or filled pauses such as “um” and “er”), and talkativeness (rate of talk per minute). Indeed, these five variables display different developmental courses in the general population^43^ and different variables distinguish different ID conditions^6^. Using these individual variables as outcome measures will be useful when interest is testing a treatment that is hypothesized to have different effects across different neural systems and learning mechanisms and thus different dimensions of language. Thus, there is a need to understand the test–retest reliability, construct validity, etc. of outcome measures reflecting various conceptually distinct dimensions of expressive language skill.

At the same time, however, the five variables we have investigated, while conceptually distinguishable, are moderately to highly correlated within any given ID condition^9,37^. Moreover, there are likely to be biomedical and/or experiential treatments that will have small but meaningful effects on multiple dimensions of language. In these latter cases, composites that summarize performance across different dimensions of language may be preferable for detecting hypothesized treatment effects. Composites can potentially increase the statistical efficiency of clinical trials, resulting in higher event rates and increased statistical precision. In turn, this can result in clinical trials that include fewer participants, are less costly, and can be completed more quickly. Composites are also useful to meet the requirements of many regulatory agencies that only one primary outcome measure be specified for a treatment trial. In the present study, therefore, we reanalyzed our previous ELS samples, creating empirically derived composites of the five variables and examining the psychometric properties of the composites for individuals with FXS and individuals with DS.

The primary goal of the present study was to continue addressing the need for adequate outcome measures for use in pharmacological and behavioral treatment studies focusing on individuals with ID. In particular, the study was designed (1) to empirically derive composite scores from the manualized and highly scripted version of the ELS procedures and (2) to determine whether those composite scores are psychometrically adequate as outcome measures for treatment studies. In addressing the latter aim, we examined the composite scores in terms of their test–retest reliability and construct (convergent and discriminant) validity. Composite scores were derived and evaluated separately for individuals with FXS and individuals with DS given the possibility that different composites could be optimal for each of these ID conditions.

## Methods

### Participants

Participants were recruited at five university sites across the U.S. through university research registries, clinics, and local and national parent and advocacy groups. We recruited individuals between the chronological ages (CAs) of 6 and 23 years at first testing. We adopted age 6 years as a minimum based on previous studies documenting the limited capacity of children with ID younger than 6 years to complete our ELS procedures^35,44^, as well as expectations regarding their relatively low probability of producing non-imitative multi-word utterances, their limited experience in completing table-based tasks in non-play interactions with an adult, and their relatively infrequent inclusion in clinical trials. The upper limit of 23 years was selected largely to decrease the possibility of significant cognitive decline and Alzheimer’s Disease-related dementia in the DS sample. A *t* test for independent samples indicated that the groups did not differ significantly in CA (see Table 1).

**Table 1:** Characteristics of participants by diagnostic group.

Measure	Fragile X syndrome			Down syndrome		
	M	SD	Range	M	SD	Range
Chronological age	15.77	4.19	6.84 to 23.76	16.86	4.56	7.60 to 23.72
NVIQ^a^	47.13^c^ (49.62)	6.45 (13.34)	42–68 (13.72 to 76.31)	48.84^f.^ (52.66)	6.89 (11.43)	42–66 (25.96 to 77.10)
VIQ^a^	49.06^d^ (50.06)	7.53 (13.49)	42–78 (17.50 to 83.85)	46.12^g^ (42.48)	5.77 (13.30)	43–72 (7.43 to 73.03)
Full scale IQ^a^	45.68^e^ (49.92)	6.88 (12.43)	40–66 (19.06 to 74.64)	45.03^h^ (47.62)	6.02 (11.89)	40–67 (23.93 to 75.07)
ADOS severity^b^	5.46^e^	2.58	1 to 10	3.19^g^	2.18	1–9

For FXS, *n* = 80 unless indicated otherwise. For DS,* n* = 78 unless indicated otherwise.

^a^Stanford-Binet, 5th edition standard scores. Values in parentheses are the Sansone et al. deviation IQs. ^b^Severity Score from Autism Diagnostic Observation Schedule, 2nd edition; ^c^*n* = 78; ^d^*n* = 79; ^e^*n* = 78; ^f^*n* = 73; ^g^*n* = 74; ^h^*n* = 73.

Participants also met the following criteria (according to parent/guardian report): (1) spoken language is the primary means of communication; (2) produces at least occasional three-word or longer utterances; (3) English is the primary language of the home; (4) no worse than a mild hearing loss; and (5) no uncorrected visual impairments serious enough to preclude meaningful engagement in the testing battery. Participants also were required to have IQs of 70 or less, according to parent report and record review at study entry and confirmed through direct testing by the project team. We also required confirmation via medical records of the *FMR1* full mutation (i.e., CGG repeats > 200) in the case of FXS and a trisomy 21 karyotype in the case of DS.

The full sample consisted of 106 individuals with FXS and 107 individuals with DS, with details of the samples provided in^9,37^, respectively. In the present study, we included only those individuals who were fully compliant (see^9,37^ for operational definition) on both the initial testing and the retesting for both conversation and narration (*n* = 80 for FXS and *n* = 78 for DS). For the combined FXS and DS sample of the present study, the racial/ethnic distribution was white (70%), African American/Black (9%), Asian/Pacific Islander (1%), Hispanic (11%), multiple races/ethnicities (6%); and Other (2%). As expected, the number of females was less than the number of males in the FXS sample but relatively equal in the case of the DS sample (19 of 80 and 43 of 78, respectively).

Once enrolled, we excluded or tried to reschedule any participant who had a change in a behavior-controlling medication (e.g., SSRIs) or in behavioral therapy/educational programming less than 8 weeks before the first visit or between the first and retest visits. These changes were documented through parent report. See^37^ for more details.

This study was approved by the Institutional Review Board at each of the five participating universities of the authors (Emory University, Rush University, University of Arizona, University of California, Davis, University of Wisconsin-Madison). Informed written consent was obtained from the parent/legal guardian prior to participation and assent was obtained from each participant. The authors affirm that all procedures contributing to this work complied with the ethical standards of the relevant national and international committees on human experimentation and with the Helsinki Declaration of 1975, as revised in 2008, and all relevant local and federal regulations.

### Measures

The measures reported on here are a subset of a larger battery of direct assessments, questionnaires, and interviews. The full battery was administered over the course of one or two days depending on the stamina of the participant, although scheduling and logistical challenges required administering the battery over a longer time frame for a few participants. The interval from beginning to end of testing was never more than eight days for any given participant.

### Participant description measures

The following measures were administered on an individual basis to participants with the aim being to characterize their degree of impairment.

#### Intellectual functioning

The Stanford-Binet Intelligence Scales, Fifth Edition (SB-5;^45^) were administered. The SB-5 is appropriate for the age range of 2–89 + years. We administered the 10 subtests that together yield a nonverbal, verbal, and full-scale IQ. Each subtest has a mean IQ = 100 and SD = 15 in the norming sample. These scores were used to determine eligibility for the study and provide a description of the participants (see Table 1). Because many participants achieved the lowest score possible on the SB-5, we also computed deviation IQs following the procedures outlined by Sansone et al.^27^.The means (and SDs) for the deviation IQs are also presented in Table 1. *t* tests for independent samples indicated that the verbal IQ scores were significantly higher for the participants with FXS than for the participants with DS for both the standard score, *t*(145.41) = 2.72, *p* < 0.01 (two-tailed), and the deviation score, *t*(145) = 3.42, *p* < 0.001 (two-tailed). No other comparisons between the diagnostic groups were significant. Note that lack of participant compliance or examiner error (e.g., failure to establish a ceiling) led to missing values for a few participants.

#### ASD symptom severity

The Autism Diagnostic Observation Schedule, 2nd edition (ADOS-2;^46^). The ADOS-2 is comprised of activities that create an opportunity to observe behaviors reflective of the core impairments of autism. The ADOS-2 has five modules, each designed for individuals with different degrees of impairment and verbal skills. The module for each participant was selected according to the manual guidelines. Because of the level of ID and relative lack of independence reported, none of the participants in this study met guidelines for administration of Module 4 or the Toddler Module. The ADOS-2 was administered and scored by research-reliable examiners. Severity scores are presented in Table 1, with higher scores reflective of more severe symptoms. Errors in administration, participant noncompliance, and scheduling difficulties resulted in missing values for a small number of participants. t tests for independent samples indicated that the severity scores were significantly higher for the participants with FXS than for the participants with DS, *t*(139) = 5.60, *p* < 0.001 (two-tailed).

### Expressive language sampling

Expressive language samples were collected twice from each participant (test and retest), with a target interval of four weeks between the two administrations. Deviations from the 4-week window reflected scheduling difficulties and accommodation to treatment changes. At each time point, samples were taken in two contexts—conversation and narration—following the procedures outlined by Abbeduto et al.^37^, with the order of administration randomized across participants. Alternate forms of conversation and narration (A and B) were administered. Participants who received version A at the initial test, received the alternate version at retest. Assignment to version at the initial test was random. All examiners who administered the ELS procedures completed a standardized training process to ensure fidelity of administration^37^. In both conversation and narration, examiner behavior was scripted to limit the amount of examiner talk and the extent to which the examiner prompted or scaffolded the participant’s talk so that variation among participants in their talk reflected differences in their expressive language skills rather than differences in examiner behavior. Conversation and narrative samples were audiorecorded in quiet testing rooms with the examiner and participant seated at a table, and the relative positions of examiner, participant, and digital recorder, and the recorder settings were standardized. Manuals describing in detail the procedures for ELS administration, training, and the assessment of fidelity are available at: https://ctscassist.ucdmc.ucdavis.edu/ctscassist/surveys/?s=W9W99JLMNX.

#### Conversation

The conversation task consists of an interview-style interaction with the examiner that is designed to encourage the participant to talk as much as possible in a 12-min period. The examiner relies primarily on open-ended prompts to topics (e.g., “Tell me everything you did at school yesterday) and broad follow-up questions and prompts (e.g., “What do you like about school”?), while minimizing their own participation. Sessions begin with a topic (based on parent report) of interest to the participant (e.g., “I was talking with your mom, and she told me that you love going on nature walks. That sounds very interesting. Tell me about that.”). After no more than 3 min (and typically less), the examiner moves from this idiosyncratic topic to the first in a list of predetermined topics in a standard order. The goal is to introduce at least three topics from the list. The examiner relies largely on open-ended questions and prompts to encourage talk without constraining the amount or nature of the talk the participant produces. Additional details of the conversation materials and procedures can be found in the online manual referenced previously. Only the first 10 min of the conversation are analyzed.

#### Narration

In the narration task, the participant is asked to tell the story depicted in a wordless picture book. The participant first looks at each page spread of the book for ~ 10 s per page without talking so as to get a sense of the story. The examiner controls the page turning. The participant then tells the story page by page. The examiner controls the book and waits 5–7 s after the participant has finished talking before turning the page. The examiner’s prompts and responses are standardized and limited largely to the first pages of the book. These initial prompts are designed to encourage the child to start the story but without influencing the nature or amount of talk the participant produces once they begin the narrative. There is no set time limit. Two books, each including 16 pages of story content, from the Mercer Mayer’s “Frog” series were used: *Frog Goes to Dinner* (Version A) and *Frog on His Own (*Version B). The participant’s entire narrative (on the second viewing) is transcribed.

#### Transcription and outcome measures

All ELS sessions were digitally audio-recorded and then transcribed and analyzed by the lead university site using the Systematic Analysis of Language Transcripts (SALT) software^47^. Talk was segmented into Communication-units (C-units); the upper bound of which was an independent clause and any modifiers, which could include subordinate and embedded clauses^37^. Transcription entailed a first draft by a primary transcriber, feedback from a secondary transcriber, and final editing by the primary transcriber, who after consideration of the feedback (and with discussion with the secondary transcriber if needed) decided on the changes needed. Transcribers were blind to individual participant results for other measures.

Transcribers were trained with the requirement that they achieve agreement with a gold standard transcription of a conversation and a narration from a typically developing child, an individual with FXS, and an individual with DS, with different a priori levels of agreement established for different dimensions of the transcription process (e.g., segmentation to C-units, number of morphemes). We evaluated inter-transcriber agreement for 22 completed samples (10 DS and 12 FXS) for the larger project by comparing transcripts prepared independently by two different teams (i.e., a different primary and secondary transcriber). Inter-transcriber agreement averaged 89% for the FXS samples and 84% for the DS samples across relevant dimensions of the transcription process. Complete inter-transcriber agreement data are provided for DS and FXS in^9,37^, respectively. It should be noted that these levels of inter-transcriber agreement have been previously found to be adequate for achieving high rates of agreement (reflected in intraclass correlations exceeding 0.80, *p* ≤ 0.005) between independently completed transcriptions in terms of the five dependent variables of interest in this study^37^. Further details on transcription, transcriber training, and inter-transcriber agreement, can be found in^9,37^. In addition, the full transcription manual is freely available at: https://ctscassist.ucdmc.ucdavis.edu/ctscassist/surveys/?s=TF9MJACKMPJMF3R4.

We focused on the five ELS outcome measures evaluated in our previous psychometric studies^9,35,37^ computing each separately for conversation and narration. Each measure was computed automatically by SALT or with minimal transformation of SALT-generated variables (e.g., the proportion for the unintelligibility measure). Descriptive statistics for each ELS-derived measure are reported in Tables 2 and 3 for the participants with FXS and the participants with DS, respectively. The measures were:

**Table 2:** Means and standard deviations for ELS-derived variables for participants with FXS.

Measure	Conversation				Narration			
	Visit 1		Retest		Visit 1		Retest	
	M	SD	M	SD	M	SD	M	SD
Lexical diversity	79.76	29.60	83.49	31.11	70.33	31.26	72.28	31.74
Syntax	3.69	1.31	3.84	1.35	4.96	2.14	5.12	2.21
Talkativeness	14.91	5.44	15.02	4.91	12.18	5.54	11.97	5.01
Unintelligibility	0.14	0.12	0.14	0.13	0.15	0.17	0.15	0.15
Dysfluency	0.21	0.13	0.21	0.12	0.18	0.12	0.20	0.14

**Table 3:** Means and standard deviations for ELS-derived variables for participants with DS.

Measure	Conversation				Narration			
	Visit 1		Retest		Visit 1		Retest	
	M	SD	M	SD	M	SD	M	SD
Lexical diversity	80.32	32.48	83.65	32.79	69.74	33.32	73.27	33.60
Syntax	3.65	1.55	3.78	1.62	4.84	2.02	4.97	2.18
Talkativeness	13.49	3.83	13.43	3.39	9.55	3.60	10.19	3.58
Unintelligibility	0.23	0.17	0.23	0.18	0.20	0.19	0.21	0.19
Dysfluency	0.27	0.15	0.28	0.14	0.25	0.15	0.27	0.16

##### Lexical diversity

This variable reflects the size of the participant’s expressive vocabulary and is operationalized as the number of different word roots in the first 50 complete and fully intelligible C-units in the sample (or the full sample of complete and fully intelligible C-units if fewer than 50 C-units). Higher scores reflected greater maturity.

##### Syntax

We computed the mean length of C-unit measured in morphemes (MLU) for complete and fully intelligible C-units. This measure provides a coarse measure of expressive syntactic competence, reflecting the fact that many syntactic achievements that occur during language development result in the production of longer utterances in terms of number of morphemes (e.g., acquisition of markers for tense and number, for passive voice, and for integrating multiple clauses into a single utterance through coordination or subordination;^48,49^). At the same time, not all increases in length will reflect greater syntactic complexity and not all new syntactic acquisitions result in increases in length. Despite its limitations, however, this measure is well accepted in the field of language development and disorders as a suitable proxy for the level of syntactic development achieved by an individual^50–52^. Higher scores on this measure reflected greater maturity.

##### Unintelligibility

This variable reflects speech articulation problems and is computed as the proportion of the total C-units that are either partly or fully unintelligible in transcription. Note that in deciding whether a stretch of unintelligible speech was one or more than one C-unit, transcribers relied on intonation and pauses; if there were no clear intonational cues or pauses, the stretch was considered a single C-unit. Higher scores reflected less maturity.

##### Talkativeness

Talkativeness was operationalized as the number of C-units attempted per minute and was intended to reflect the motivation to talk. Note that this is not intended to reflect rate of articulation as would be the case if we computed mean duration (in seconds) of C-unit; instead, our operationalization reflects the amount of talk contributed by the participant. Although the amount of talk could have been characterized in terms of syllables, words, or other linguistic elements rather than C-units, there is precedent in the field for using C-units^53^. Moreover, there is reason to expect variables reflecting these different operationalizations to be at least moderately correlated. We have found in previous research that although this talkativeness variable captures individual differences in engagement and the proclivity to participate in linguistic interaction, it is not related to variations in developmental level for the general population, at least not between the ages of 3.5 years and young adulthood^43^.

##### Dysfluency

We computed the proportion of the total number of complete and fully intelligible C-units that included one or more maze, or verbal dysfluency (e.g., um, uh, er, or repetition of word parts or words, or a revision of a portion of the C-unit). This variable has been previously shown to index various aspects of utterance planning (e.g., selection of syntactic elements) and thus, to be highly dependent on working memory^54–56^. Although over the life course, we would expect a decrease in the proportion of dysfluent utterances, we have found previously that as learners continue to master the various aspects of language, but before that learning is fully consolidated, dysfluency scores are actually *positively* correlated with measures of, for example, lexical diversity and syntax^9,37^. Studies of typically developing children have yielded similar findings, suggesting that dysfluencies in developing populations increase with age (and developmental level) because of the speaker’s inexperience in planning utterances with newly acquired linguistic elements and patterns^57–59^. Thus, in the ability range of our present sample of participants, we expected higher dysfluency scores to reflect greater maturity.

### Construct validity measures

We administered a battery of standardized tests and informant-report measures to capture the same dimensions of expressive language skill assessed with the ELS procedures to assess convergent validity. We also included a measure that we hypothesized assessed largely different skills than ELS as a way of evaluating discriminant validity. For any given participant, ELS examiners were blind to the performance on the construct validity measures. Missing data on these measures resulted from scheduling difficulties, participant noncompliance, and examiner errors.

#### Convergent validity

We used two subtests from the *Clinical Evaluation of Language Fundamentals-4*^*th*^* edition* (CELF-4)^60^, which is an individually administered standardized test designed for ages 5 through 21 years. The CELF-4 *Formulated Sentences* (FS) subtest was administered. In the FS subtest, a participant is asked to generate a full sentence about a visual stimulus that incorporates a target word or phrase provided by the examiner. The FS subtest was designed to measure expressive syntactic ability, and so, would be expected to correlate with the ELS syntax measure. The *Expressive Vocabulary* (EV) subtest was also administered. In the EV subtest, the participant is asked to generate the names for pictured people, objects, and actions. The EV subtest was designed to assess the breadth of expressive vocabulary and thus, it was expected to correlate with ELS lexical diversity. To provide a developmental ability score across the full age-range of participants comparable to the ELS scores, we used raw scores (i.e., number correct) for each CELF-4 subtest as many participants with ID will score at floor in terms of standard scores.

The *Verbal Working Memory* subtest of the *Stanford-Binet-5*^*th*^* edition* (SB-5 VWM) was administered. This subtest was designed to measure the ability to store and manipulate verbal information and plan a verbal response. The easy items from the SB-5 VWM require the immediate and exact repetition of phrases and sentences, and the more difficult items require recalling the last words of questions that have been answered previously. Here too, we used raw scores from the SB-5 VWM subtest to provide an ability score comparable to the ELS scores and avoid floor effects common for people with ID who complete the SB-5 VWM^27^. As already noted, there is evidence that dysfluencies increase in frequency during development as speakers grapple with planning utterances with newly acquired linguistic elements and patterns. At the same time, improvements in verbal working memory provide the foundation for those acquisitions^61–63^. Thus, the SB-5 VWM subtest score was expected to correlate positively with the dysfluency variable.

The *Goldman-Fristoe Test of Articulation, 2*^*nd*^* edition* (GFTA-2;^64^) Sounds in Words (SiW) subtest was administered to assess speech articulation and was expected to correlate negatively with the ELS unintelligibility measure. The GFTA is designed for ages 2 through 21 years. In the GFTA-2, the examiner presents stimuli for the participant to label. points to a picture and says a target word, which the participant is expected to repeat. All samples were audio-recorded using digital recorders and scored at the lead university site. Following the procedures outlined in the GFTA-2 manual, every target response was scored in terms of whether each of the target sounds was pronounced correctly or incorrectly. Responses were not transcribed. Those who scored the administrations were familiar with the GFTA-2 procedures and highly experienced in working with individuals with DS and individuals with FXS. Although not required by standard manualized procedures, each participant’s testing session was scored independently by two scorers, with any discrepancies in scoring between the coders reviewed and resolved by a third scorer; all instances requiring a third coder involved review by either a Master’s-level speech-language clinician or a PhD-level developmental psychologist with a background in language development and neurodevelopmental disabilities (AJT). We used the percentage of correct phonemes produced in the GFTA-2 single-word imitation procedure to provide an ability score comparable to the ELS score.

The *Vineland Adaptive Behavior Scales, Second Edition* (VABS-II;^65^) was completed as a self-administered questionnaire by the parent/guardian. In the VABS-II, the parent/guardian rates the participant’s mastery of everyday functional skills in the domains of socialization, daily life skills, and communication. The VABS-II was normed for ages 3–21 years. We calculated the raw score for the Expressive Communication (EC) domain to obtain a general measure of communication skill and the motivation to engage in communication. The VABS-II has been widely used for research and clinical diagnosis in the field of ID. We expected this VABS-II EC score to correlate positively with ELS talkativeness.

#### Discriminant validity

The VABS-II Maladaptive Behavior Index (VABS-II MBI;^65^) was used to establish the discriminant validity for the ELS composites. This measure was designed to assess the parent/guardian’s perception of the participant’s internalizing, externalizing, and other challenging behaviors that could impede successful adaptive functioning. MBI total raw score was used in the present analyses.

### Statistical analysis

#### Primary analyses

We conducted a principal components analysis (with varimax rotation) to empirically guide our creation of the ELS composites. A separate analysis was conducted for the FXS and DS samples. In each analysis, we included all 10 ELS-derived variables (5 from conversation and 5 from narration). Following the principal components analyses, we created the composites by first transforming all variables into *z* scores and then taking the mean of the variables that loaded most highly in terms of magnitude on a component to create the composite representing that component.

We conducted parametric analyses to examine the psychometric properties of each ELS composite: Pearson correlations and intraclass correlations to establish test–retest reliability and Pearson correlations to establish construct validity. In each of these parametric analyses, we corrected for multiple tests using the false discovery rate (FDR) procedure of Benjamini and Hochberg^66^, thereby controlling the false discovery rate at 5%; however, we also present the uncorrected *p* values to provide additional information to eventual users of these outcome measures. All *p* values are for two-tailed tests. Note that our previous work with stratified subsets of participants in the larger study^37^, suggested that we had adequate power for detecting at least moderate to large correlations.

#### Supplementary analyses

We have suggested that arriving at a comprehensive characterizatin of expressive language skills and changes in those skills requires administering and analyzing both conversational and narrative samples^44^. The reason for this recommendation is that the two contexts have advantages and disadvantages. For example, narration tends to elicit more complex syntax than does conversation and thus, the former is best for determining the upper bounds of an individual’s syntactic competence. In contrast, conversation tends to elicit more diverse vocabulary than narration and thus, the former is better suited for assessing lexical breadth. Nonetheless, there may be logistical or mechanistic reasons to choose only a single context in a treatment study. Thus, we conducted supplementary analyses designed to replicate the main analyses but for composites derived separately for conversation and narration.

## Results

Descriptive statistics for the five ELS-derived variables in conversation and narration are presented separately for the two diagnostic groups in Tables 2 and 3. Although not explicitly of interest in the present study, the differences between diagnostic groups and ELS context are largely consistent with previous research. For example, unintelligibility scores were significantly higher for the participants with DS than for those with FXS in both conversation (*t*[132.77] = 3.69, *p* ≤ 0.001) and narration (*t*[151.83] = 2.06, *p* ≤ 0.050), and syntactic scores were higher in narration than conversation for both diagnostic groups (*t*[79] = 7.11, *p* > 0.001 for FXS; (*t*[77] = 8.62, *p* > 0.001 for DS). At the same time, however, some differences relative to previous findings occurred. For example, the diagnostic groups did not differ in their syntactic scores in either conversation (*t*[150.72] = 0.17, *p* ≥ 0.050) or narration (*t*[156]  = 0.36, *p* ≥ 0.050). Note, however, that this latter finding may reflect the fact that we included here only those participants who were fully compliant on both the test and retest administrations of both ELS contexts and thus, the participants in the present study were in some sense among the more competent of their diagnostic group peers.

The principal components analysis for the participants with FXS yielded three components that collectively explained 77.59% of the total variance in the set of 10 ELS-derived variables. Based on the magnitude of the loadings (see Table 4), *Composite 1* was defined by the lexical diversity, syntax, and dysfluency variables from conversation and narration; *Composite 2* was defined by the conversation and narration unintelligibility variables; and *Composite 3* was defined by the conversation and narration talkativeness variables.

**Table 4:** Principal components analysis loadings of ELS variables by diagnostic group.

ELS variable	Principal components		
	Composite 1	Composite 2	Composite 3
Fragile X syndrome			
Dysfluency-con	**0.885**	− 0.125	− 0.022
Syntax-con	**0.868**	− 0.256	0.151
Lexical diversity-con	**0.864**	− 0.201	0.164
Talkativeness-con	0.100	0.197	**0.846**
Unintelligibility-con	− 0.221	**0.843**	0.163
Dysfluency-nar	**0.740**	− 0.155	− 0.086
Syntax-nar	**0.697**	− 0.492	− 0.189
Lexical diversity-nar	**0.673**	− 0.497	0.267
Talkativeness-nar	− 0.017	− 0.071	**0.919**
Unintelligibility-nar	− 0.272	**0.846**	− 0.017
Down syndrome			
Dysfluency-con	0.291	**0.840**	− 0.171
Syntax-con	**0.787**	0.387	0.129
Lexical diversity-con	**0.801**	0.392	0.102
Talkativeness-con	0.121	− 0.227	**0.842**
Unintelligibility-con	− **0.815**	− 0.038	0.135
Dysfluency-Nar	0.204	**0.833**	− 0.042
Syntax-nar	**0.843**	0.382	0.005
Lexical diversity-nar	**0.820**	0.273	0.317
Talkativeness-nar	− 0.069	0.041	**0.881**
Unintelligibility-nar	− **0.782**	− 0.039	0.092

*n* = 80 (FXS), *n* = 78 (DS). Bolded values were those that loaded most highly on the component and thus, these are taken as the variables defining the component.

The principal components analysis for the participants with DS also yielded three components that collectively explained 77.15% of the total variance in the set of 10 ELS-derived variables. However, the variables loaded somewhat differently compared to the FXS participants. Based on the magnitude of the loadings in Table 4, *Composite 1* was defined by the lexical diversity, syntax, and unintelligibility variables from conversation and narration; *Composite 2* was defined by the conversation and narration dysfluency variables; and *Composite 3* was defined by the conversation and narration talkativeness variables.

The composite scores for the FXS and DS samples were then created by taking the mean of the Z-scores for the variables defining each component per participant in the respective diagnostic group (with reverse scoring of the Z scores for unintelligibility before summing for Composite 1 for the DS participants). Separate composite scores were created for the initial and retest administrations of the ELS procedures. The bivariate correlations and intraclass correlations between the test and retest composites are shown in Table 5 for the participants with FXS and in Table 6 for the participants with DS. The bivariate correlations between the composites at the initial administration and the standardized language measures are shown in Tables 7 and 8 for the participants with FXS and the participants with DS, respectively.

**﻿Table 5:** Test–retest reliability: bivariate correlations and intraclass correlations.

ELS composite	Fragile X syndrome	
	r	Icc^a^
Composite 1^b^	0.91****	0.95****
Composite 2^c^	0.82****	0.81****
Composite 3^d^	0.77****	0.87****

Uncorrected *p* values for individual tests are marked with asterisks as follows: ****p ≤ 0.001. Bold cells contain values significant at p ≤ 0.050 after FDR correction for multiple tests.

^a^Mixed model, assuming no interaction, and absolute agreement.

^b^Defined by high loadings for lexical diversity, syntax, and dysfluency in conversation and narration.

^c^Defined by high loadings for unintelligibility in conversation and narration.

^d^Defined by high loadings for talkativeness in conversation and narration.

**﻿Table 6:** Test–retest reliability: bivariate correlations and intraclass correlations.

ELS composite	Down syndrome	
	r	ICC^a^
Composite 1^b^	0.84****	0.91****
Composite 2^c^	0.82****	0.90****
Composite 3^d^	0.71****	0.83****

Uncorrected *p* values for individual tests are marked with asterisks as follows: ****p ≤ 0.001. Bold cells contain values significant at p ≤ 0.050 after FDR correction for multiple tests.

^a^Mixed model, assuming no interaction, and absolute agreement.

^b^Defined by high loadings for lexical diversity, syntax, and unintelligibility in conversation and narration.

^c^Defined by high loadings for dysfluency in conversation and narration.

^d^Defined by high loadings on talkativeness in conversation and narration.

**Table 7:** Convergent construct validity: participants with fragile X syndrome.

Measures	CELF-4 EV	CELF-4 FS	VABS-II EC	GFTA-2 SiW	SB-5 VWM
Composite 1^a^	0.49****	0.71****	0.36****	0.50****	0.64****
Composite 2^b^	− 0.27**	− 0.50****	− 0.34****	− 0.64****	− 0.47****
Composite 3^c^	− 0.01	− 0.02	0.04	− 0.05	− 0.07

Uncorrected *p* values for individual tests marked with asterisks as follows: *p ≤ 0.05, **p ≤ 0.01, ****p ≤ 0.001. Bold cells contain values significant at p ≤ 0.050 after FDR correction for multiple tests.

^a^Defined by high loadings for lexical diversity, syntax, and dysfluency in conversation and narration.

^b^Defined by high loadings for unintelligibility in conversation and narration.

^c^Defined by high loadings for talkativeness in conversation and narration.

**﻿Table 8:** Convergent construct validity: participants with down syndrome.

Measures	CELF-4 EV	CELF-4 FS	VABS-II EC	GFTA-2 SiW	SB-5 VWM
Composite 1^a^	0.52****	0.51****	0.41****	0.51****	0.56****
Composite 2^b^	0.30**	0.19	0.35***	0.40****	0.26*
Composite 3^c^	0.12	0.18	− 0.15	− 0.09	0.05

Uncorrected *p* values for individual tests marked with asterisks as follows: *p ≤ 0.050, **p ≤ 0.01 ***p ≤ 0.005, ****p ≤ 0.001. Bold cells contain values significant at p ≤ 0.050 after FDR correction for multiple tests.

^a^Defined by high loadings for lexical diversity, syntax, and unintelligibility.

^b^Defined by high loadings for dysfluency in conversation and narration.

^c^Defined by high loadings for talkativeness in conversation and narration.

In terms of test–retest reliability, Tables 5 and 6 show that all bivariate and intraclass correlations were significant in the case of both groups of participants, even after application of the FDR procedure. In computing the intraclass correlations, we report results for a mixed model, assuming no interaction, and absolute agreement. Thus, the composites show strong test–retest reliability.

In terms of convergent construct validity, Table 7 illustrates that, for the participants with FXS, *Composite 1* was correlated significantly with all the standardized measures. *Composite 2* was correlated significantly with all the standardized measures except the CELF-4 EV (after application of the FDR). In contrast, *Composite* 3 did not correlate significantly with any of the standardized measures. Table 7 also shows that, for the participants with FXS, the highest correlation in terms of absolute magnitude was with the CELF-4 FS subtest in the case of *Composite* 1 and with the GFTA-2 SiW subtest in the case of *Composite 2*. Thus, there was strong evidence of convergent construct validity for the composite defined by the lexical diversity, syntax, and dysfluency variables and the composite defined by the unintelligibility variables.

For the participants with DS, *Composite 1* was correlated significantly with all the standardized measures (see Table 8). *Composite 2* was correlated (after correction via the FDR procedure) with the Vineland EC and the GFTA-2 SiW subtest. As was true for the FXS sample, *Composite 3* did not correlate significantly with any of the standardized measures for the participants with DS. Thus, there was strong evidence of convergent construct validity for the composite defined by the lexical diversity, syntax, and unintelligibility variables but somewhat weaker evidence of convergent validity for the composite defined by the dysfluency variables.

In terms of discriminant validity, none of the bivariate correlations between the ELS composites and the VABS-II MBI was statistically significant for either diagnostic group. The correlations ranged from -0.04 to 0.16 for the participants with FXS and from -0.07 to 0.16 for the participants with DS.

### ﻿Supplementary analyses

The results of the supplementary analyses are presented in supplementary Tables S1–S6. In the case of both participants with DS and participants with FXS, the first principal component was defined by high positive loadings for vocabulary, syntax, and dysfluency, as well as a high negative loading for unintelligibility. This was true for both conversation and narration as well. The only minor exception was that for the FXS participants, unintelligibility also had a high positive loading (along with talkativeness) for the second composite in conversation. Nonetheless, we examined the psychometrics only for the composite derived from the Z scores of the variables of the first principal component because of the lack of construct validity evidence for talkativeness. As seen in the supplementary tables S1–S6, the composites for conversation and for narration were each associated with strong test–retest reliability as well as strong evidence supporting convergent and divergent construct validity.

## Discussion

The present study was designed to address the pressing need for psychometrically adequate outcome measures for use in pharmacological and behavioral treatment studies involving individuals with ID. The study builds on previous research that has (1) documented the feasibility of scripted ELS procedures for collecting conversational and narrative data from individuals with FXS or DS and (2) evaluated the psychometric properties of five variables derived from those procedures^9,35,37^. In the present study, we focused on composite scores empirically derived from the ELS variables. Composite scores are attractive for studies in which the treatment of interest is expected to have pervasive effects across multiple domains of language or in which the treatment is expected to have an effect on language, but the specificity of its effects is unknown. In either case, a composite score could be expected to provide a more robust measure of treatment efficacy than would any variable indexing only a single domain of language.

Using principal component analysis, we found that the 10 ELS-derived variables (five from conversation and five from narration) clustered into three components, or composite scores. Interestingly, however, the composite scores are defined by somewhat different variables for the participants with FXS and those with DS. In the case of FXS, (1) the lexical diversity, syntactic, and dysfluency variables defined one composite. The remaining two composites for the participants with FXS each reflect a singular domain of language; namely, (2) unintelligibility in conversation and narration and (3) talkativeness in conversation and narration. In the case of DS, the three composite scores were defined by (1) the lexical diversity, syntactic, and unintelligibility variables, (2) the dysfluency variables, and (3) the talkativeness variables. These findings suggest the need to tailor the selection of language composite scores to the ID condition of interest. More generally, consistent with theory and considerable previously published empirical data, these findings underscore the possibility that the mechanisms leading to language challenges are partly different across different etiological conditions causing ID.

We also examined the composite scores in terms of their short-term test–retest reliability and construct validity. Consistent with our previous results for the individual ELS-derived variables^9,35,37^, all the composite scores demonstrated strong test–retest reliability over a four-week administration interval, and this was true for both the participants with FXS and those with DS. This is a critical requirement for an outcome measure. In the absence of intervention or any expectation for naturally occurring age- or time-related change, an individual’s performance should be consistent from one time to the next in terms of its absolute level and standing relative to peers. All the composite scores derived demonstrated this consistency, both for the participants with FXS and those with DS, demonstrating the potential utility of the composite scores for treatment studies, as well as for characterizing change more generally (e.g., in a natural history study).

At the same time, however, not all the composite scores were supported in terms of construct validity. In particular, the composite defined by talkativeness in both conversation and narration, which emerged for both etiological groups, did not correlate with any of the standardized tests or the informant report measure used to establish convergent validity. In many respects, this is not a surprising finding as it is similar to previous work examining the talkativeness measures separately for conversation and narration^9,37^. Thus, combining the two talkativeness measures into a single composite did not increase their association with the external validation measures. It is possible, however, that the constructs measured by the external validation measures we chose, including the VABS-II EC subtest, simply did not overlap sufficiently with the talkativeness construct. In any event, we must conclude that the ELS talkativeness measure, as a operationalized in the present study, is not recommended for treatment studies at this time.

Note, however, that other operationalizations of talkativeness are possible. For example, one could compute the proportion of C-units produced by the participant relative to the proportion of all C-units produced in the sample (i.e., the participant’s C-units plus the partner’s C-units). That operationalization was not selected in the present study because the standardization of the examiner’s talk in the procedures used for conversation and narration reduced the amount of, and variability in, examiner talk. In other, less constrained, sampling procedures, a proportion of talk relative to other participants might be useful. Even with our ELS procedures, however, it could be useful in the future to use words or other linguistic units rather than C-units to quantify the amount of talk, although as noted previously all such variations are likely to be correlated.

The other composites, however, generally were supported by strong evidence of convergent validity and discriminant validity. For the participants with FXS, the composites defined by the composite scores of (1) lexical diversity, syntax, and dysfluency and (2) the unintelligibility variables, respectively, were each significantly correlated with virtually all the external validity measures. For the participants with DS, the composite defined by lexical diversity, syntax, and unintelligibility was correlated with all external validity measures and the composite score defined by the dysfluency variables was correlated with two of the five external validity measures. The measure chosen to evaluate discriminant validity (i.e., the VABS-II MBI) was not correlated with any of the composite scores. Thus, there are at least two composite scores that can be derived from ELS procedures that are promising from a psychometric perspective for use in treatment studies in FXS and two in DS.

It is interesting to note that the conversation and narrative versions of each variable aligned with the same component in the principal component analysis. This finding is consistent with the results of previous psychometric studies^9,37^, which have found significant correlations between the measures derived from the two sampling contexts. In addition, we found in our supplementary analyses that if only a single context is used, the best composite is one that includes the syntax, vocabulary, dysfluency, and unintelligibility measures. Moreover, these analyses suggested that this composite has strong test–retest reliability and strong evidence of construct validity. These findings raise the possibility that one or the other sampling context can be omitted if there is a concern with the testing burden on participants. At same time, however, it has been demonstrated in several previous studies that the two contexts “pull” for different levels of performance in some domains of language. Thus, it is important to balance decisions about which sampling context(s) to use not only on testing burden but also on the hypothesized language domains of greatest interest.

It is important to note several limitations of the present study. First, the participants ranged in age from 6 to 23 years of age and spontaneously used multiword utterances at least occasionally. Generalizing the findings beyond this age and ability range is thus not warranted. In addition, we included in the sample for the present study only those participants who were highly compliant (i.e., meaningfully completed both the initial and retest administration of both conversation and narration). Again, cautions about generalizability are thus necessary. It also remains to be seen whether the composites are sensitive to change. Second, although many aspects of the sampling and recording contexts were standardized, there were factors that could not be completely controlled and that could influence some of the measures. For example, the unintelligibility measure could be influenced by variations between and within participants in speaking volume or in the direction they faced when speaking. Controlling all such sources of variation, however, might not be practical or desirable for ensuring generalizability of findings to real-world linguistic interactions. Third, it is important to note that most norm-referenced standardized tests, including those we used in our construct validity analyses, were designed for purposes of identification or diagnosis of language or other developmental problems and not for use as outcome measures in treatment studies. Moreover, we did not assess the test–retest reliability of these tests within our study. Consequently, it should not be concluded from the present study that ELS is superior to these tests as an outcome measure in general. As we pointed out previously, however, there are several reasons to prefer ELS for deriving outcome measures, including enhanced generalizability to real-world communicative contexts and low testing burden on participants. Fourth, the five measures we have computed are quite coarse, proving only a broad summary of an individual’s skills in the domain of interest. Our measure of syntax (i.e., MLU), for example, correlates with achievements in many aspects of syntax, from mastery of inflectional morphology to use of various forms of clause embedding. At the same time, however, MLU does not capture all aspects of syntactic development, nor does it provide guidance on areas of syntax most in need of remediation for an individual. Expansion to more detailed and nuanced measures within language domains should thus, be a focus on future research.

More generally, the ELS procedures studied were created for English speakers, and thus, additional work in terms of translation and cultural appropriateness is needed, although this work has begun for speakers of Spanish (see^67^). The resources needed for transcription also remain a barrier for use in multisite, large-scale treatment studies; however, we are exploring ways of reducing the transcription burden. Data are also needed on the sensitivity of the composite scores to (naturally occurring or treatment induced) change, although there have been several studies documenting the utility of some of the individual ELS variables for characterizing naturally occurring longitudinal change in several populations.

Despite the limitations noted, the present study substantially advances the goal of providing psychometrically adequate outcome measures for testing the efficacy of treatments for individuals with ID. We have identified useful composites for treatment studies involving people with two common forms of ID. In the case of FXS, a useful general expressive language composite would be focused on vocabulary, syntax, and planning problems, whereas in the case of DS, a composite reflecting vocabulary, syntax, and speech articulation problems would be suggested.

## Supplementary Information

Supplementary Tables.

## Data Availability

The datasets used and/or analyzed for the present paper can be made available upon a reasonable request to the corresponding author.
