# Supplementary material for: Expressive language sampling and outcome measures for treatment trials in fragile X and down syndromes: composite scores and psychometric properties
Source: Sci Rep. 2023 Jun 7;13:9267. doi: 10.1038/s41598-023-36087-3 (PMC10247708; doi:10.1038/s41598-023-36087-3)
Supplement: Supplementary file 1 — Supplementary Tables. [file 41598_2023_36087_MOESM1_ESM.docx]

Supplementary Tables

Supplementary Table 1. Principal Components Analysis Loadings by ELS Context for Participants with Fragile X Syndrome

| **ELS Variable** | **Principal Components** | |
| --- | --- | --- |
| **Conversation** | | |
|  | **Composite 1** | **Composite 2** |
| Dysfluency | **.880** | -.092 |
| Syntax | **.953** | -.005 |
| Lexical Diversity | **.933** | .027 |
| Talkativeness | .194 | **.881** |
| Unintelligibility | **-.470** | **.645** |
| **Narration** | | |
|  | **Composite 1** | **Composite 2** |
| Dysfluency | **.743** | -.101 |
| Syntax | **.888** | .156 |
| Lexical Diversity | **.858** | .345 |
| Talkativeness | -.016 | **.987** |
| Unintelligibility | **-.734** | -.056 |
| *n* = 80. Bolded values were those that loaded highly on the component and thus, these taken as taken as the variables defining the composite. | | |

Supplementary Table 2. Principal Components Analysis Loadings by ELS Context for Participants with Down Syndrome

| **ELS Variable** | **Principal Components** | |
| --- | --- | --- |
| **Conversation** | | |
|  | **Composite 1** | **Composite 2** |
| Dysfluency | **.653** | -.485 |
| Syntax | **.943** | .075 |
| Lexical Diversity | **.948** | .046 |
| Talkativeness | .065 | **.953** |
| Unintelligibility | **-.714** | .064 |
| **Narration** | | |
|  | **Composite 1** | **Composite 2** |
| Dysfluency | **.639** | -.091 |
| Syntax | **.914** | -.030 |
| Lexical Diversity | **.893** | .295 |
| Talkativeness | -.031 | **.987** |
| Unintelligibility | **-.776** | .098 |
| *n* = 79. Bolded values were those that loaded highly on the component and thus, these were taken as taken as the variables defining the composite. | | |

Supplementary Table 3. Test-Retest Reliability for Composites by ELS Context for Participants with Fragile X Syndrome

| **ELS Composite** | **Index of Reliability** | |
| --- | --- | --- |
|  | ***r*** | ***ICC***^a^ |
| **Conversation Composite^b^** | .84**** | .92**** |
| **Narration Composite^b^** | .87**** | .93**** |
| Uncorrected *p* values for individual tests are marked with asterisks as follows: *****p* < .001. Shaded cells contain values significant at p < .050 after FDR correction for multiple tests (separately for *r*s and *ICC*s).  ^a^Mixed model, assuming no interaction, and absolute agreement. ^b^Defined by high loadings for lexical diversity, syntax, dysfluency, and unintelligibility. | | |

Supplementary Table 4. Test-Retest Reliability for Composites by ELS Context for Participants with Down Syndrome

| **ELS Composite** | **Index of Reliability** | |
| --- | --- | --- |
|  | ***r*** | ***ICC***^a^ |
| **Conversation Composite^b^** | .87**** | .93**** |
| **Narration Composite^b^** | .87**** | .93**** |
| Uncorrected *p* values for individual tests are marked with asterisks as follows: *****p* < .001. Shaded cells contain values significant at p < .050 after FDR correction for multiple tests (separately for *r*s and *ICC*s).  ^a^Mixed model, assuming no interaction, and absolute agreement. ^b^Defined by high loadings for lexical diversity, syntax, dysfluency, and unintelligibility. | | |

Table 5. Convergent and Discriminant Construct Validity for Composites by ELS Context for Participants with Fragile X Syndrome

| **Measures** | **CELF-4**  **EV** | **CELF-4**  **FS** | **VABS-II**  **EC** | **GFTA-2**  **SiW** | **SB-5**  **VWM** | **VABS-II**  **MBI** |
| --- | --- | --- | --- | --- | --- | --- |
| **Conversation Composite^a^** | .48**** | .65**** | .33*** | .56**** | .57**** | -.03 |
| **Narration Composite^a^** | .43**** | .72**** | .42**** | .56**** | .68**** | -.09 |
| Uncorrected *p* values for individual tests marked with asterisks as follows: **p* < .05, ****p* < .005, *****p* < .001. Shaded cells contain values significant at p < .050 after FDR correction for multiple tests (computed separately for conversation and narration and for convergent and discriminant validity). ^a^Defined by high loadings for lexical diversity, syntax, dysfluency, and unintelligibility. | | | | | | |

Supplementary Table 6. Convergent and Discriminant Construct Validity for Composites by ELS Context for Participants with Down Syndrome

| **Measures** | **CELF-4**  **EV** | **CELF-4**  **FS** | **Vineland-II**  **EC** | **GFTA-2**  **SiW** | **SB-5**  **VWM** | **Vineland-II**  **MBI** |
| --- | --- | --- | --- | --- | --- | --- |
| **Conversation Composite^a^** | .61**** | .52*** | .56**** | .68**** | .64**** | -.03 |
| **Narration**  **Composite^a^** | .69**** | .56**** | .48**** | .65**** | .66**** | .07 |
| Uncorrected *p* values for individual tests marked with asterisks as follows: **p* < .05, *****p* < .001. Shaded cells contain values significant at p < .050 after FDR correction for multiple tests. ^a^Defined by high loadings for lexical diversity, syntax, dysfluency, and unintelligibility. | | | | | | |
